# The Potential for Bioactive Peptide Production in a Fermented Dairy Beverage Based on Chickpea Water Extract Using Proteolytic Lactic Acid Bacteria

**DOI:** 10.3390/foods15122249

**Published:** 2026-06-22

**Authors:** Mahboobeh Ahangaran, Mahmood Gharaviri, Ivan A. Fomenko, Irina Chernukha, Leonid I. Kovalev, Dmitry A. Kulikov, Natalia G. Mashentseva

**Affiliations:** 1 Department of Biotechnology and Bioorganic Synthesis, Russian Biotechnological University (ROSBIOTECH), Volokolamskoye Highway, 11, 125080 Moscow, Russia; garavirim@mgupp.ru (M.G.); iv.fomenko@mail.ru (I.A.F.); kulikovda@mgupp.ru (D.A.K.); natali-mng@yandex.ru (N.G.M.); 2 Experimental Clinic and Research Laboratory for Bioactive Substances of Animal Origin, V.M. Gorbatov Federal Research Center for Food Systems, Talalikhina St., 26, 109316 Moscow, Russia; imcher@inbox.ru; 3 A.N. Bach Institute of Biochemistry of the Russian Academy of Sciences, Leninsky Prospekt, 33c3, 119071 Moscow, Russia; likovalyov@rambler.ru

**Keywords:** plant-based drink, fermentation, chickpea, peptides, proteomic methods

## Abstract

A chickpea-based milk beverage containing both plant and animal proteins represents an excellent substrate for the production of biologically active peptides through fermentation. Fermentation by lactic acid bacteria (LAB) increases its nutritional value compared to the unfermented beverage while improving the digestibility and bioavailability of essential nutrients via proteolytic enzyme activity. This study investigated the production of bioactive peptides in fermented chickpea water extract using ten bacterial strains isolated from plant and animal sources. The proteolytic activity of each strain was quantified using the trinitrobenzene sulfonic acid (TNBS) method, and the presence of proteolytic genes was confirmed via agarose gel electrophoresis. Peptides released during fermentation were identified through two-dimensional electrophoresis, matrix-assisted laser desorption/ionization time-of-flight mass spectrometry (MALDI-TOF MS), and tandem mass spectrometry. To predict the potential biological activities of the studied peptide sequences, a series of in silico analyses were performed using specialized bioinformatics tools. The identified peptides were predicted to exhibit antioxidant, antihypertensive, anticancer, antibacterial, antifungal, antituberculosis, and angiotensin-converting enzyme (ACE) inhibitory activities. Based on the results, *L. fermentum* SB-2 and *L. sakei* SD-8, were selected as promising candidates for bioactive peptide production in a chickpea water extract-based milk beverage and were subsequently applied in the beverage prototype.

## 1. Introduction

These days, plant-based drinks are becoming more popular due to health concerns associated with dairy (such as lactose intolerance and allergies) and the growing demand for plant-based diets, making them a significant market for the food industry [1]. Additionally, plant proteins are thought to be a good source of bioactive peptides, which may have applications in functional diets to improve health and prevent disease. These peptides, known as bioactive peptides (BAPs), display a variety of biological activities after being released, including angiotensin-converting enzyme (ACE) inhibition, antioxidant, antibacterial, and anticancer effects [2].

Bioactive peptides can be released from protein sources through several pathways, including enzymatic or chemical hydrolysis, digestion in the gastrointestinal tract, and specific food processing methods like fermentation with proteolytic starter cultures [3]. Recently, peptides derived from fermented plant materials, especially legumes, which contain protein, have received much attention. Their production occurs through endogenous plant proteases and microbial proteolytic systems during fermentation [4].

Lactic acid bacteria (LAB) are one of the most important types of bacteria in the food industry due to their non-pathogenic nature and suitability for technological and industrial processes [5]. These bacteria are Gram-positive and acid-tolerant, and their morphology can be rod-shaped, coccus, or coccobacillus. They exhibit key metabolic properties, such as proteolytic activity (protein hydrolysis), and have been widely used as starter cultures in food fermentation for many years [6]. Fermentation is a process where microorganisms (e.g., bacteria) are cultivated on a protein substrate, using their endogenous enzymes (e.g., proteases) to hydrolyze it into shorter peptides and amino acids. The extent of hydrolysis depends on factors such as the microbial strain, protein source, fermentation conditions (pH, temperature, time), and enzyme activity [7].

LAB possess complex proteolytic systems, including cell envelope proteases (CEPs), peptide transporters, and intracellular peptidases for optimal growth and hydrolysis of proteins into free amino acids and oligopeptides in nutrient-limited environments (such as milk or fermented foods), [8]. Several types of cell envelope proteases (CEPs) have been identified in LAB, including PrtP from *Lactobacillus lactis*, PrtR from *Lactobacillus rhamnosus*, PrtB from *Lactobacillus bulgaricus*, PrtH from *Lactobacillus helveticus* and PrtS from *Streptococcus thermophilus* [9]. As part of this proteolytic system, casein is hydrolyzed into oligopeptides by an external cell envelope-associated proteinase (CEP), peptides are taken up by specific transport systems inside the cell, and a variety of intracellular peptidases convert peptides into amino acids. Additionally, during fermentation, this proteolytic system may release bioactive, health-beneficial peptides that help fermented foods develop their flavor and texture [10].

One of the most popular legumes in the world, chickpeas are a complete meal that can be prepared in a variety of ways and are regarded as one of the most affordable, safe, and high-quality sources of dietary protein. Along with a distinct amino acid profile that includes lysine, tyrosine, glutamic acid, and histidine, chickpeas also contain diverse bioactive compounds that protect human health. Chickpeas are high in dietary fiber, low in fat and sodium, and a great source of vitamins, minerals, and folate. They are also a great source of complex carbohydrates [11]. Research shows that protein fermentation of legumes, especially chickpeas, produces peptides that can improve human health [12]. Products made from processing chickpeas are frequently utilized in the meat, dairy, confectionery, bakery, and other food industry sectors to determine the final product’s texture and consistency. It should be mentioned that the biological value and functional qualities of chickpea seeds’ proteins dictate their suitability for various food industry applications [13].

Recent studies have provided ample evidence of the degradation of chickpea proteins, especially globulins and albumins, into smaller bioactive peptides through enzymatic hydrolysis and fermentation [14]. These hydrolyzed peptides are a major field of study for nutrition and health scientists since they have a number of advantageous bioactivities. According to studies, globulins make up between 53 and 60% of the total protein in chickpeas, making them the main protein type [15]. The nutritional value of chickpeas is greatly enhanced by these proteins, which are abundant in important amino acids. Along with other health-promoting properties like anti-inflammatory action, the hydrolysis of chickpea proteins produces peptides that have been shown to have antibacterial, antioxidant, antihypertensive, and angiotensin-converting enzyme (ACE) inhibitory activity [16,17,18,19].

While soybean and pea proteins have been extensively studied as substrates for LAB-mediated peptide generation, chickpea (*Cicer arietinum* L.) offers distinct advantages: its globulin-rich protein profile (53–60% of total protein) contains unique sequences rich in lysine, tyrosine, and glutamic acid that may yield novel bioactive peptides upon proteolysis [15,20]. Additionally, chickpea has naturally lower levels of certain anti-nutritional factors compared to soybean and is inherently gluten-free, broadening its applicability. Despite these advantages, research on LAB fermentation of chickpea water extract—a minimally processed, cost-effective substrate—remains limited, representing a specific knowledge gap this study aims to address.

The development of plant-based beverage enriched with bioactive compounds, particularly fermentation-derived bioactive peptides known for their health benefits, has gained significant research attention. The aim of this study is to utilize bacterial strains carrying the proteinase gene for controlled fermentation of chickpea water extract, produce a fermented dairy beverage, and characterize the resulting bioactive peptides via proteomics. The identified peptides will then be analyzed using in silico approaches to predict their potential biological activities. To achieve this goal, the study was designed in two sequential phases: (1) screening of proteolytic lactic acid bacteria strains for their ability to ferment chickpea water extract and release bioactive peptides; and (2) application of the most promising strains to develop a fermented dairy beverage prototype containing both plant and animal proteins (55% cow’s milk + 45% chickpea extract), in compliance with Eurasian Economic Union regulations for milk-containing products.

## 2. Materials and Methods

Lactic acid bacteria were isolated from different natural materials to produce biologically active peptides in chickpea beverage. Chickpeas (*Cicer arietinum* L.) of the kabuli type used in this experiment were obtained from the local market in Iran.

### 2.1. Microorganisms, Media, and Growth Conditions

In this experiment, bacterial strains were isolated from different sources (Table 1). 25 g of each sample was homogenized in 250 mL of sterile saline solution (0.85% NaCl, pH 7.0). Then, 10 mL dilutions were prepared from each sample and cultured on MRS agar (HiMedia Laboratories Pvt. Ltd., Maharashtra, India). The plates were incubated for 48 h at 37 °C under anaerobic conditions. Colonies were chosen for further growth on MRS broth and agar until complete isolation based on phenotypic traits such as circular shape, creamy texture, white, gray, or green color.

Bacterial strains were identified by MALDI-TOF MS using a Bruker Biotyper system (Bruker Daltonics, Manning Park Billerica, MA, USA). Protein fingerprint spectra were acquired in the mass range of 2000–20,000 Da and compared against the Bruker Biotyper Reference Library for species-level identification (Supplementary Figure S1).

Based on this method, ten strains of lactic acid bacteria were identified from these natural materials (Figure S1). Lactic acid bacteria strains, including *Limosilactobacillus fermentum* SB-2, *Latilactobacillus sakei* SD-8, *Levilactobacillus brevis* VY-1, *Pediococcus pentosaceus* FC-9, *Pediococcus pentosaceus* FC-10, *Leuconostoc mesenteroides* FM-4, *Lactiplantibacillus plantarum* PC-7, *Leuconostoc mesenteroides* CH-5, *Limosilactobacillus fermentum* AC-3, *Lacticaseibacillus paracasei* CA-6 were incubated at 37 °C in MRS broth (HiMedia, India) medium. Bacterial cells were collected by centrifugation (5000× *g*, 10 min, 4 °C). Before being used, they had been washed and suspended in sterile phosphate buffer.

### 2.2. Molecular Determination and Proteolytic Activity of Selected Natural Isolates DNA Extraction

Extraction of DNA from microorganisms was done using glass beads [21]. The glass beads were preliminarily washed with sulfuric acid (100%) 1 time, then washed with distillate and dried at 60 °C. The resulting culture from a Petri dish was taken to Eppendorf with a microbiological loop and dissolved in 500 μL of lysis buffer with the following composition: 100 mM Tris HCl (pH = 8); 50 mM EDTA; 1% SDS. Approximately 400 µL (0.3 g) of glass beads were added to the resulting mixture and vigorously stirred for 3 min and allowed to stand statically for 10 min. The supernatant was transferred to a new Eppendorf with 275 µL of 7M CH3COONH4 (pH 7.0) and incubated for 5 min at 65 °C followed by another 5 min at 4 °C. Chloroform (500 μL) was added and vigorously stirred for 1–3 min. Centrifuged for 2 min at 13,000 rpm. After centrifugation, the upper layer was taken into a new eppendorf with the addition of 1 mL of 99.8% ethanol and kept for 30 min at a temperature of −20 °C. centrifuged for 10 min at 13,000 rpm at 4 °C. The precipitate was then washed with 70% ice-cold ethyl alcohol. The precipitate was dried for 5 min at 65 °C. Then the precipitate was dissolved in 30–50 µL of TE buffer or distilled water.

### 2.3. Polymerase Chain Reaction (PCR)

PCR was performed using an Eppendorf Mastercycler Gradient thermocycler (Eppendorf, USA). The list of primers used in this work is presented in Table 2. The total duration of the program was 90–140 min. PCR amplification conditions were as follows: initial denaturation at 94 °C for 4 min; 30 denaturation cycles at 94 °C, 1 min; annealing at 53–56 °C depending on the Tm (Melting Temperature) of primers (1 min); elongation at 72 °C (1.5 min) and final elongation at 72 °C for 7 min.

Electrophoresis of DNA samples was carried out in a horizontal electrophoresis chamber SE-2 (Helicon) in 1% agarose gel. Then the Samples were mixed with 6× Loading Dye Solution (6× LD) prior to loading. GeneRuler 1 kb DNA Ladder (Fermentas, Lithuania) was used as a marker. Gels were visualized and documented using a BioRad Gel-Doc system. Before preparative electrophoresis, pure buffer was poured into the phoresis chamber, and after the completion of the process, the gel was placed in a new solution of ethidium bromide (0.5 μg/mL). The results obtained were analyzed for the presence of the necessary amplification fragments.

### 2.4. Preparation of Chickpea Water Extract and Fermentation

The preparation of chickpea water extract and its fermentation was done according to the method of Zhang [22], with a slight modification. 50 g of chickpea seeds were washed with distilled water and then 250 mL of sterile distilled water was added to it and soaked for 10 h at 25 °C. After homogenizing the chickpea solution, it was filtered with double-layer gauze to separate the insoluble residues and boiled at 100 °C for 15 min to sterilize and inactivate the enzymes. To increase the growth of microorganisms, 1% glucose was added to the samples. After cooling the chickpea water extract to room temperature, lactic acid microorganisms were added to the amount of 1.2 × 10^8^ CFU/mL (based on McFarland’s turbidity standard) (1:20) and incubated for 72 h at 37 °C. The sample was aseptically collected at 0, 24, 36 and 72 h, immediately cooled on ice, and stored at −70 °C until analysis.

#### Formulation of Fermented Dairy Beverage

Recently, innovative food products combining both animal and plant-derived ingredients have been increasingly developed to promote healthier lifestyles. Despite the nutritional advantages of plant proteins, their pronounced flavor and limited solubility restrict their widespread application in the food industry. To overcome these techno-functional limitations, combining plant proteins with dairy proteins has emerged as a promising strategy to enhance plant protein utilization while minimizing negative impacts on sensory properties, improving nutritional value, and reducing ingredient costs [23,24]. Previous studies have explored similar hybrid products with varying ratios of chickpea extract to cow’s milk (90:10, 70:30, 50:50, 30:70, and 10:90), with the 30:70 and 50:50 ratios proving most successful [20].

In accordance with the technical regulations of the Eurasian Economic Union for milk-containing products (TR CU 033/2013), which require a minimum of 20% milk solids in the dry matter of the final product, a hybrid formulation containing 55% (*v*/*v*) pasteurized cow’s milk (0.5% fat) and 45% (*v*/*v*) chickpea water extract was selected. Based on the Phase 1 screening results, *L. fermentum* SB-2 and *L. sakei* SD-8, which exhibited the highest proteolytic activity and favorable sensory properties, were chosen as starter cultures.

The beverage was prepared by homogenizing the milk-chickpea mixture, followed by pasteurization at 85–87 °C for 10 min. After cooling to 37 °C, the mixture was inoculated with the selected bacterial consortium at an initial concentration of 10^7^ CFU/mL. Fermentation was carried out at 37 °C for 24 or 72 h, followed by rapid cooling to 4 ± 2 °C and storage for up to 21 days prior to analysis.

### 2.5. Quantification of Free Alpha Amino Groups Using (TNBS) Method

The measurement of amino groups plays an important role in many biological sciences. Numerous techniques already in use for this aim rely on the aminic nitrogen’s nucleophilic properties. Among these, techniques utilizing nitrobenzenic derivatives such 2,4,6-trinitrobenzene-1-sulfonic acid (TNBS)1 and 1-fluoro-2,4-dinitrobenzene (1–3) are highly well-known. The final chemical is unique to fundamental amino groups. This technique has a very broad use in food technology and determines the amount of free alpha-amino groups, which indicates protein hydrolysis and proteolytic activity [25,26].

In this experiment, free amino acids in the supernatant of a chickpea water extract fermented with lactic acid bacteria were measured using 2,4-6-trinitrobenzenesulfonic acid (TNBS, Sigma-Aldrich, Saint Louis, MO, USA) [26]. The reaction with TNBS was carried out in 15 mL Falcon tubes. 2 mL of 0.2125 M sodium phosphate buffer, pH 8.20, 200 µL of 1% SDS solution, 50 µL of the prepared sample, and 2 mL of a freshly prepared 0.1% aqueous solution of TNBS were sequentially added to the falcon tube. 2 mL of 0.2125 M sodium phosphate buffer (pH 8.20), 250 µL of 1% sodium dodecyl sulfate solution and 2 mL of freshly prepared 0.1% solution of TNBS were added to the falcon tube with a blank sample. 2 mL of 0.2125 M sodium phosphate buffer (pH 8.20), 250 µL of the standard solution, and 2 mL of freshly prepared 0.1% TNBS solution were mixed to falcon tubes with different concentrations of the standard.

L-leucine (Sigma-Aldrich, USA) was used as the standard. A stock solution of L-leucine (3.0 mmol/L in 1% SDS) was diluted in 1% aqueous SDS to prepare a series of standards with concentrations ranging from 0.15 to 3.0 mmol/L. The Falcon tubes containing the experimental, blank, and standard samples were tightly sealed with screw caps, briefly vortexed (PV1 vortex, Grant Bio, Devizes, UK; 10 s), and then incubated in a GFL water bath (Germany; 50 ± 1 °C, 1 h) with an opaque lid. After incubation, 4.0 mL of 0.1 M HCl was added to each tube to terminate the reaction.

The Falcon tubes were tightly sealed with screw caps, vortexed (PV1, Grant Bio, UK; 10 s) and then allowed to cool at room temperature for 30 min. For optical density (OD) measurements 200 µL of each solution (in triplicate) was transferred to a 96-well flat-bottom UV-transparent microplate (UV-Star, Greiner Bio-One, Frickenhausen, Germany). The OD at 340 nm was measured using a Synergy 2 microplate reader (BioTek, Shoreline, WA, USA).

**Figure 1:**
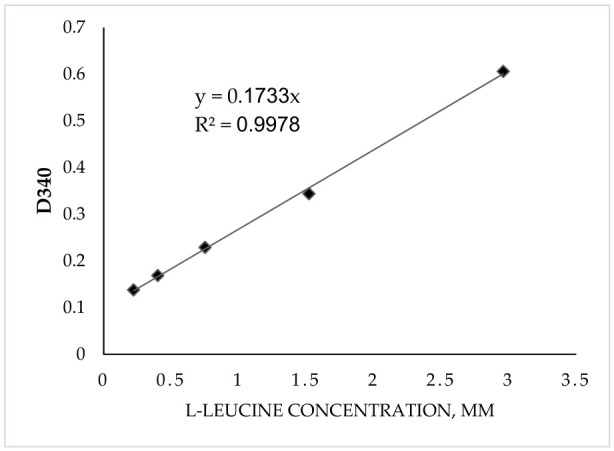
Calibration curve for the dependence of optical density on the concentration of L-leucine in the reaction mixture. The black squares represent the experimental data points (mean values of triplicate measurements) for the L-leucine standard solutions.

A calibration curve (Figure 1) was used to quantify the results, expressed in mmol/L (or mmol/dm^3^) of L-leucine equivalents.

### 2.6. Two-Dimensional Electrophoresis (2DE)

Two-dimensional electrophoresis, following the method described by O’Farrell [27], was used to identify proteins. This involved isoelectric focusing in ampholytes (IEF-PAGE, equilibrium type) to establish isoelectric points. Protein detection on two-dimensional electropherograms was performed by sequential staining with Coomassie Blue R-250 (CB R-250) followed by silver nitrate. For computer densitometry, wet two-dimensional electropherograms were used. Their complete digital images (or selected fragments) were obtained by scanning on an Epson Expression 1680 scanner at 300 dpi resolution in 48-bit color, with results saved in TIFF format. The resulting digital images were processed in a graphics editor, and protein quantification was performed using Image Master 2D Platinum v7.0 (GE Healthcare, Glattbrugg, Switzerland). Two-dimensional electrophoresis was performed in triplicate for each sample (*n* = 3 biological replicates). Spot detection and quantification were performed using identical parameters across all gels. Normalization was achieved by expressing each spot’s volume as a percentage of the total spot volume in the respective gel. Only spots present in all three replicates with a coefficient of variation (CV) < 20% were considered for further analysis.

### 2.7. MALDI-TOF MS and MS/MS Analysis

Matrix-assisted laser desorption/ionization time-of-flight mass spectrometry (MALDI-TOF MS) was used to accomplish peptide mass fingerprinting. In accordance with Zvereva [28], protein spots were removed from two-dimensional electrophoresis gels, destained, and followed by in-gel tryptic digestion. The resulting peptide mixtures were analyzed by MALDI-TOF MS for mass fingerprinting, followed by tandem MS (MS/MS) to confirm the peptide sequence, increasing the confidence of protein identification [29,30]. A MALDI-Ultraflex TOF/TOF equipment (Bruker, Ettlingen, Germany) with a 336 nm UV laser and positive ion mode was used for the mass spectrometric analysis. Trypsin autolysis peaks (*m*/*z* 842.51 and 2211.10) were used for internal calibration, and spectra obtained over a mass range of 500–8000 Da were used. Standard bioinformatics methods, which include Mascot (version 2.4) [Matrix Science, USA] for database searching, were used to process the obtained peptide mass fingerprints.

For the identification of short peptides naturally released during fermentation, sample suspensions were centrifuged at 800× *g* for 5 min, and the supernatant fraction, diluted 50-fold with distilled water, was directly analyzed by MALDI-TOF MS and tandem MS/MS without prior enzymatic digestion. This workflow specifically targets peptides generated by bacterial proteolytic activity during fermentation, excluding artifacts from in-gel tryptic digestion.

Peptide bioactivity prediction was performed using specialized databases: BIOPEP-UWM, AntiCP, AHTpin, AntiBP, AntiFP, AntiTb, and ToxinPred. Prediction confidence thresholds were set as follows: BIOPEP-UWM (probability ≥ 0.75 or presence of validated motifs), AntiCP/AHTpin/AntiBP/AntiFP/AntiTb (probability ≥ 0.65), and ToxinPred (toxicity probability < 0.5). All sequences were cross-validated against the NCBI database using Mascot. It is important to note that these in silico tools rely solely on primary amino acid sequences and do not account for gastrointestinal digestion stability, intestinal absorption, or synergistic effects within complex food matrices. Therefore, reported activities should be interpreted as preliminary computational indicators requiring experimental validation.

### 2.8. Consumer Sensory Acceptance

Sensory evaluation was designed as a preliminary screening to assess strain-dependent acceptability rather than a full-scale consumer study. A panel of 30 untrained university students (20–25 years old), all regular consumers of dairy or plant-based beverages, participated in the study. Samples (24 h and 72 h fermented beverages) were presented in a randomized order and served at 10 ± 2 °C in 50 mL portions. Participants rated appearance, aroma, taste, texture, and overall acceptability on a 9-point hedonic scale (1 = strongly dislike; 9 = strongly like). Water and unsalted crackers were provided for palate cleansing between samples. Data were analyzed using paired t-tests with significance set at *p* < 0.05. As an untrained panel, this assessment provides an initial indicator of product feasibility and strain suitability; comprehensive sensory profiling with trained assessors is recommended for future validation. The product complied with TR CU 021/2011 (Food Safety Regulation). According to Russian National Standard GOST R 52379-2005 and Federal Law No. 323-FZ, formal ethical approval is mandatory only for clinical trials of medicinal products. This study was a non-interventional sensory evaluation of a safe food product, not a clinical trial. All participants were adults, gave informed consent, could withdraw at any time, and their data were anonymized.

### 2.9. Acid-Forming Activity Experiment

To ascertain the acid-producing activity of a dairy product containing chickpea water extract, titratable acidity (TA) and pH were assessed using standard analytical methods. A pH meter (ANION-4100) was used to measure the sample’s pH after ten milliliters were allowed to equilibrate at room temperature (25 °C). The International Dairy Standard Method (IDF 74B:2004) was also used to measure the titratable acidity of a 0.1 N NaOH solution using phenolphthalein as an indicator. This acidity was converted from Turner’s degree (°T) to percent lactic acid equivalent using the following formula: %Lactic Acid = 0.009 × °T, °T = VNaOH × 10.

### 2.10. Approximate Chemical Composition Measurement

The protein, fat, ash and carbohydrate content of the milk beverage containing chickpea water extract was determined according to standard methods (AOAC, 2019).

Total solids (dry matter) were determined by drying approximately 5 g of the sample at 105 °C to constant weight in a forced-air oven (AOAC Method 925.23). The protein content of the samples was determined using the Kjeldahl method (AOAC Method 990.03) and the total nitrogen was multiplied by 6.38 to obtain the estimated total nitrogen. The fat content was estimated using the petroleum ether extraction method (AOAC Method 989.05). The ash content of the sample was determined by combustion (AOAC Method 923.03), two grams of the sample was placed in a crucible in a muffle furnace at 550 °C for 3 h or more to obtain a light gray ash, and its initial and final weights were taken. The carbohydrate content was calculated by difference:

Carbohydrate (%) = Total solids (%) − [Protein (%) + Fat (%) + Ash (%)]

### 2.11. Statistical Analysis

All fermentation experiments were conducted in triplicate (*n* = 3 biological replicates). For spectrophotometric measurements, each sample was analyzed in triplicate (technical replicates). Data are expressed as mean ± standard deviation (SD). Paired t-tests were used to compare sensory scores between fermentation times. Pearson’s correlation coefficient was calculated to evaluate the relationship between pH and proteolytic activity. Statistical significance was set at *p* < 0.05. Analyses were performed using Statistica 10.0 (StatSoft, Tulsa, OK, USA).

## 3. Results and Discussion

### 3.1. Organoleptic Evaluation of Fermented Chickpea Water Extract

The organoleptic assessment of the fermented chickpea water extract demonstrated notable strain-dependent differences in aroma profiles, which are essential for consumer acceptance of plant-based fermented drinks. Strains such as Lactiplantibacillus plantarum PC-7, Pediococcus pentosaceus FC-9 and Limosilactobacillus fermentum SB-2 produced fresh, slightly acidic, and pleasant aromas, likely due to the production of desirable volatile organic compounds (VOCs) [31]. On the other hand, strains such as Pediococcus pentosaceus FC-10, Leuconostoc mesenteroides FM-4, and Limosilactobacillus fermentum AS-3 produced undesirable, pungent, or acidic flavors, likely attributable to elevated levels of acetic acid, sulfur compounds, or branched-chain aldehydes, which are often linked to off-flavors in fermented products [32]. Considering the importance of sensory properties, among these strains, strains *L. fermentum* SB-2, *L. sakei* SD-8, and *L. plantarum* PC-7 are the most promising candidates due to their pleasant aroma profiles (Table 3).

### 3.2. Determination of Protease Genes

**Figure 2:**
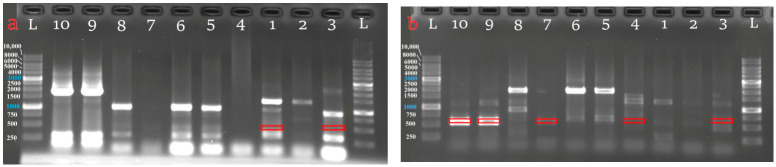

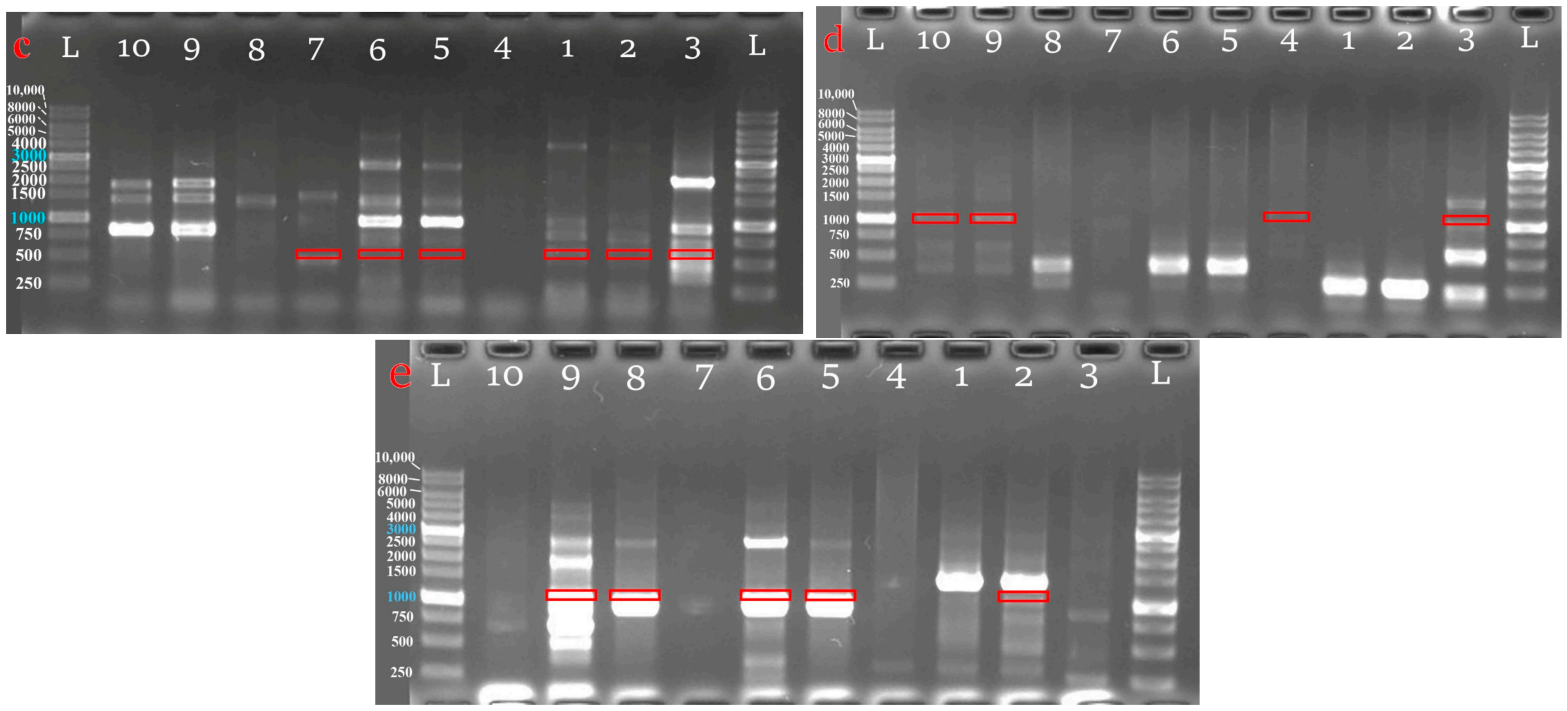
Gel electrophoresis results of PCR products amplified with primer and the presence of proteolytic genes: (**a**)—prtP ⁄prtM (685 bp) gene pair, (**b**)—prtP (560 bp), (**c**)—prtB (597 bp), (**d**)—prtH (1034 bp), (**e**)—prtR (1052 bp). L—Ladder 1 Kb DNA Ladder (DL006) (Geneaid Biotech Ltd., Taiwan), 1—*L. paracasei* CA-6, 2—*L. fermentum* AS-3, 3—*Leuc. mesenteroides* CH-5, 4—*L. plantarum* PC-7, 5—*Leuc. mesenteroides* FM-4, 6—*P. pentosaceus* FC-10, 7—*P. pentosaceus* FC-9, 8—*L. fermentum* SB-2, 9—*L. sakei* SD-8, 10—*L. brevis* VY-1. The red frames indicate the target amplicon bands of the expected sizes for each respective gene.

PCR results (Figure 2) showed that all 10 strains studied in this experiment contained protease genes, including prtM, prtB, prtH, prtR and prtP, whose proteolytic activity has been reported [9,33]. The presence of proteinase genes in each strain identified with specific primers can be seen in Table 4. The prtH gene (1034 bp) was present in fewer strains than the prtB gene (597 bp), which was more prevalent than the other genes in a broad variety of strains. Similarly, only the strains of Limosilactobacillus fermentum SB-2 and Levilactobacillus brevis VY-1 presented the prtM-prtP intergenic region (685 bp). Some strains, such as Levilactobacillus brevis VY-1 and Limosilactobacillus fermentum AS-3, had multiple proteinase genes, indicating that they had a wider range of proteolytic capabilities that might make them more effective in degrading complex proteins during fermentation. Strains with diverse proteinase gene spectra are particularly valuable for creating bioavailable fermentation products, as previous research has indicated [34].

### 3.3. Determination of Free Amino Group

The free amino groups and the degree of protein hydrolysis were measured using the TNBS method and the results were expressed as L-leucine equivalents (mM). The change in L-leucine equivalents (Δ) was calculated as the difference between these two points 0 and 72 h after fermentation, with these Δ values varying from 3.38 to 17.35 mM. These variations indicate strain-dependent differences in proteolytic activity (Table 5).

Compared with the control group, *Limosilactobacillus fermentum* SB-2, *Latilactobacillus sakei* SD-8, and *Levilactobacillus brevis* VY-1 significantly increased amino acid release. Conversely, no detectable changes were observed between the uninoculated control group and *Lacticaseibacillus paracasei* CA-6, *Lactiplantibacillus plantarum* PC-7, or *Lactiplantibacillus fermentum* AS-3 (*p* > 0.05).

**Figure 3:**
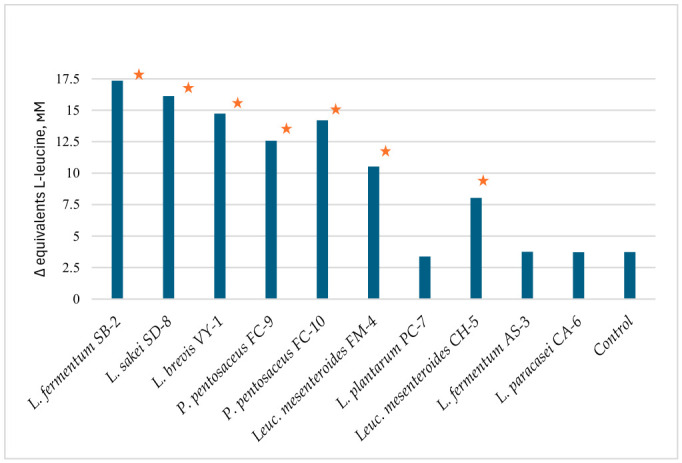
Effect of bacterial strains on protein hydrolysis of chickpea water extract. Data are expressed as mean ± standard deviation (*n* = 3). Stars indicate statistically significant differences compared to the unfermented control.

A bar plot of mean ΔL-leucine values across strains with standard deviations highlighted strain-dependent hydrolysis efficiency, with significant groups marked by asterisks (Figure 3).

The relationship between pH decrease and peptide hydrolysis (ΔL-leucine) was assessed using Pearson’s correlation coefficient (r), which quantifies the strength and direction of the linear relationship between two continuous variables. The Pearson coefficient is calculated as:

$$r=\frac{\sum_{i=1}^{n}\left(x_{i}-\bar{x})(y_{i}-\bar{y}\right)}{\sqrt{\sum_{i=1}^{n}{\left(x_{i}-\bar{x}\right)}^{2}}\cdot \sqrt{\sum_{i=1}^{n}{\left(y_{i}-\bar{y}\right)}^{2}}}$$

A high negative correlation between ΔL-leucine and pH drop was found using Pearson correlation analysis (r = −0.89, *p* < 0.001), indicating that more extensive proteolysis is linked to greater acidification.

According to the statistical results, the bacterial strain used in the experiment plays an important role in determining the degree of proteolysis during the fermentation of chickpea water extract. Strains such as *Latilactobacillus sakei* SD-8 and *Limosilactobacillus fermentum* SB-2 significantly increased the release of free amino groups, indicating that they may increase the nutritional value of the beverage.

Analysis of proteins and peptides.

#### 3.3.1. Identification of Chickpea Water Extract Proteins Before and After Fermentation

**Figure 4:**
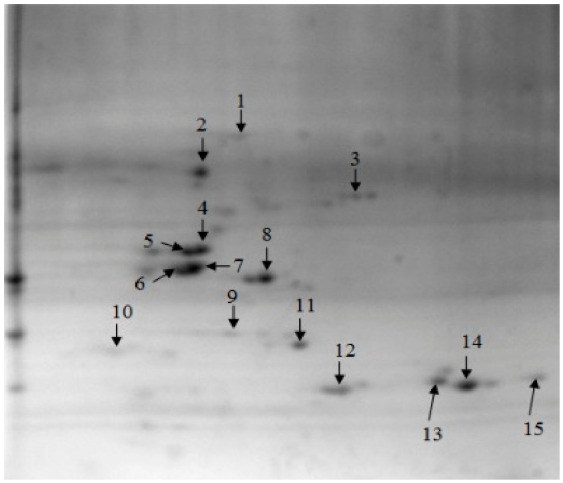
2D electrophoregram of chickpea beverage proteins (control). Stained with Coomassie R-250. Arrows with numbers indicate identified fractions in accordance with Table 6.

Two-dimensional gel electrophoresis followed by MALDI-TOF/TOF mass spectrometry was used to characterize the protein profiles of chickpea water extract. A total of 15 protein spots were identified in the control sample (Figure 4). Comparative 2-DE of chickpea water extract proteins before and after fermentation revealed distinct patterns of protein expression and degradation among the ten bacterial strains studied.

The control sample showed a consistent protein profile in which the main grain storage proteins, such as legumin and vicilin fragments, were dominant, along with biotin-containing proteins and albumin-like proteins, as identified by mass spectrometry (Table 6).

Mascot software recorded an ion score above 68 for all identified proteins, indicating a statistically significant match (*p* < 0.05). Peptide sequence coverage ranged from 20.7% to 96%, and peptide counts varied from 9 to 48 per protein, reflecting high identification confidence and spectral quality. Comparison of the isoelectric points (pI) and experimental molecular weights (Mw) with the theoretical values predicted from the amino acid sequences was performed for a more precise evaluation using ExPASy, where a moderate but statistically significant correlation was observed between the experimental and theoretical Mw. This indicates a reasonable alignment with the predicted protein migration.

**Figure 5:**
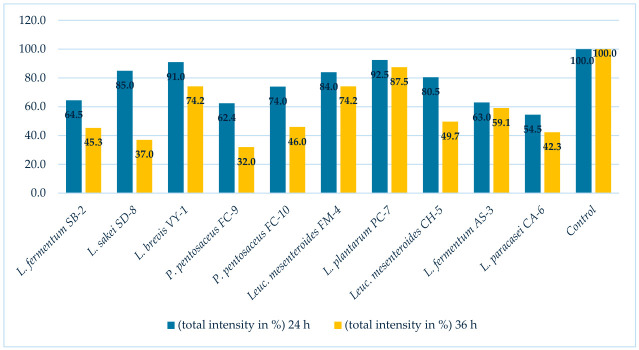
Diagram of the effect of different types of lactic acid bacteria on the change in the total amount of protein in chickpea water extract.

The computerized densitometry results of the studied samples allowed for the evaluation of the effect of different strains on total protein levels compared to the control group (Figure 5).

**Figure 6:**
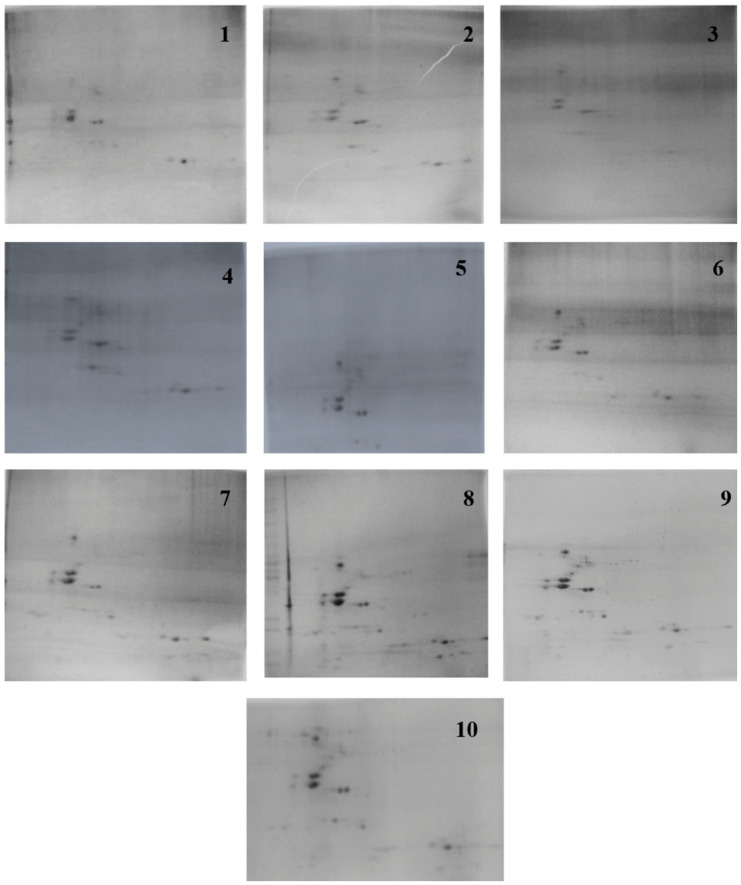
Two-dimensional electrophoresis of chickpea beverage proteins. 1—*Limosilactobacillus fermentum* SB-2; 2—*Latilactobacillus sakei* SD-8; 3—*Levilactobacillus brevis* VY-1; 4—*Pediococcus pentosaceus* FC-9; 5—*Pediococcus pentosaceus* FC-10; 6—*Leuconostoc mesenteroides* FM-4; 7—*Lactiplantibacillus planatum* PC-7; 8—*Leuconostoc mesenteriodes* CH-5; 9—*Limosilactobacillus fermentum* AS-3; 10—*Lacticaseibacillus paracasei* CA-6. Coomassie G-250 staining. after 24 h of incubation.

The maximum effect of strains on proteins was related to strain *Levilactobacillus brevis* VY-1 and the minimum effect was related to strain *Pediococcus pentosaceus* FC-9 (Figure 6).

**Figure 7:**
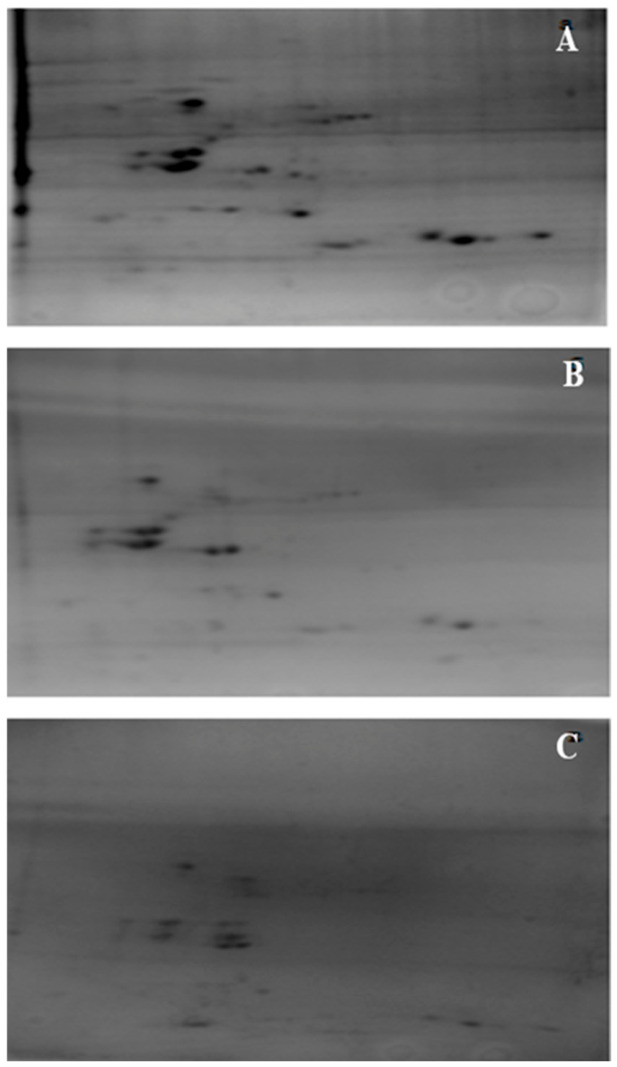
DE proteins of chickpea beverage with the maximum spread in total protein values: (**A**)—control, (**B**)—strain 3, (**C**)—strain 4 after 36 h of incubation.

In fermented samples (Figure 7), significant variation in the presence, intensity, and migration of protein spots was observed among strains, indicating different proteolytic activity. Notably, legumin A-like and vicilin-like fragments were reduced in presence or completely disappeared in several strains (particularly *L. fermentum* SB-2, *P. pentosaceus* FC-9, and *Leuc. mesenteroides* FM-4), indicating extensive protein hydrolysis. At the same time, new spots with lower molecular weight appeared in the fermented routes, particularly in *L. brevis* VY-1, *Leuc. mesenteroides* FM-4, and *Leuc. mesenteroides* CH-5, indicating the production of peptide fragments or expression of microbial proteins. These changes point to strain-specific metabolic activity that influences proteome remodeling in the chickpea matrix. These findings are consistent with previous studies reporting significant remodeling of legume proteomes during fermentation, driven by microbial enzymatic activity [35].

Revealed pI shifts in several protein spots compared to the control, suggesting potential post-translational modifications or the exposure of new charge-carrying residues following proteolysis. The extent and direction of these shifts further differentiated the strains’ impact on the protein network.

#### 3.3.2. Identification of Short Peptides (*m*/*z* up to 6000 Da) from Chickpea Water Extract

Tandem mass spectrometry (MS/MS) analysis of short peptides released during proteolysis of fermented chickpea water extract proteins by lactic acid bacteria identified 30 unique peptides at 24 h and 36 h. Table 7 details their molecular masses, amino acid sequences, parent proteins, and positions within the source protein sequence.

Mass spectra were analyzed using the Mascot program in Peptide Mass Fingerprint mode with a mass tolerance of 0.01% for MH+ ions, searching against the NCBI protein database. Bioinformatics analysis including peptide identification and bioactivity prediction was performed using specialized databases: BIOPEP (bioactive peptides), AntiCP (anticancer peptides), AntiBP (antimicrobial peptides), AHTpin (antihypertensive peptides), ToxinPred (toxicity prediction), AntiFP (antifungal peptides), and AntiTb (antitubercular peptides).

The dominant predicted activities observed in the peptide dataset were angiotensin-converting enzyme (ACE) inhibitory and anticancer (AntiCP) effects, which were present in 100% and 90% of the peptides, respectively, highlighting the in silico-predicted therapeutic potential of the peptides in the management of hypertension and cancer-related diseases (Table 8). The universal presence of ACE inhibitory activity is consistent with previous findings on legume-derived peptides and reinforces fermented chickpea as a valuable functional food matrix [36]. A substantial proportion of peptides (56.7%) also were predicted to exhibit potential antioxidative properties, suggesting their potential in combating oxidative stress.

A higher peptide diversity is observed at 24 h than at 36 h, possibly due to protein availability or optimal protease activity, as fermentation at this time maximizes peptide yield and bioactivity spectrum. Notably, no short peptides with biological activity were detected in strain *Leuconostoc mesenteriodes* CH-5.

The main precursors of bioactive peptides, according to the protein source distribution, are mostly vicilin-like and 2S-like albumin, which is consistent with previous studies [37]. Strong bioactivity profiles were also shown by sequences derived from oleosin and late embryogenesis abundant (LEA) proteins, although less often. Importantly, the mass-to-charge (*m*/*z*) values ranged from 976.5 to 4115.0, and most peptides were within the bioactive window of <3000 Da. The relatively short peptide lengths (mean ≈ 20 amino acids) support this notion, enhancing the likelihood of gastrointestinal absorption and systemic bioactivity upon consumption [38].

Bioinformatic analysis predicted seven distinct biological activities for the peptides generated by bacterial fermentation in this study. Among the tested strains, *Limosilactobacillus fermentum* SB-2 and *Latilactobacillus sakei* SD-8 exhibited superior proteolytic activity and bioactive peptide release, significantly enhancing the functional value of the fermented chickpea water extract through increased production of health-promoting compounds. In a similar study Ma [19] screened 16 bacterial strains for chickpea fermentation and identified four strains with superior proteolytic activity and peptide production from chickpea protein hydrolysis. Further support comes from Li [39], who reported that Bacillus subtilis fermentation enhanced soluble proteins, peptides, and free amino nitrogen (FAN) in chickpeas, corroborating our observations on proteolysis efficiency and antioxidant enhancement. Another report highlighted that LAB are the most valuable microorganisms for bioactive peptide production, owing to their high substrate adaptability and efficient proteolytic systems [40].

Additionally, Tangyu [41] demonstrated that fermentation of chickpea milk by LAB enhances its nutritional and sensory properties. Their work showed that LAB strains not only increased the bioavailability of L-lysine but also improved the amino acid profile. These findings are consistent with our results where our studied strains similarly increased protein digestibility. Taken together, these studies highlight the dual role of LAB in (1) improving the nutritional quality of plant-based beverages and (2) masking the bean flavor through targeted bioconversion of volatile compounds. Also, the peptides identified with different properties in this study are consistent with findings from prior reports [42,43].

### 3.4. Formulation of a Fermented Dairy Beverage Using Chickpea Water Extract

Following the strain screening phase described in Section 3.1, Section 3.2 and Section 3.3, the two most promising strains (*L. fermentum* SB-2 and *L. sakei* SD-8) were applied to develop a fermented dairy beverage prototype. The formulation (55% cow’s milk + 45% chickpea water extract) was designed to balance functional benefits, sensory acceptability, and regulatory compliance with TR CU 033/2013 for milk-containing products. Due to the poor solubility and pronounced flavor of plant proteins in human nutrition, their use in the food industry remains challenging. To solve this problem, combining these proteins with milk proteins has been proposed, which improves the organoleptic properties and nutritional value of the product as well as reduces the cost of ingredients [23,24]. Previous studies have evaluated similar hybrid products with varying milk-to-chickpea extract ratios [20].

To ensure product quality and safety in the Russian Federation, manufacturers must comply with the technical regulations of the Eurasian Economic Union (EAEU), particularly the regulations on general food safety and the composition of milk-containing products. Given the current scarcity of fermented plant-based drinks on the market and the growing global popularity of dairy-plant hybrid beverages, it was decided to develop a product meeting the EAEU requirements for milk-containing products, where the mass fraction of milk solids in the dry matter of the final product must be at least 20%. For this purpose, a recipe was formulated containing 55% cow’s milk (0.5% fat) and 45% chickpea water extract.

Based on studies, using a combination of strains is much more promising than using them separately, so the strains *L. fermentum* SB-2 and *L. sakei* SD-8, which showed great potential in producing active peptides and organoleptic properties, were selected as starter cultures for the fermentation of this milk-based herbal beverage.

#### 3.4.1. Determination of Organoleptic Properties of Beverages

One of the most crucial factors in assessing a product’s competitiveness and appeal is its organoleptic qualities. The Russian Federation’s standards state that the product must be liquid and uniform in consistency, have a distinct taste and scent of fermented milk, and be white, light cream, or creamy in color. The results of the participants’ evaluation showed that the fermented beverage after 24 h was more desirable for consumption compared to the 72 h sample. Mean taste scores were 7.5 ± 0.6 versus 5.2 ± 0.8 (*p* < 0.001), reflecting a milder sourness preferred by panelists. Similarly, appearance (7.3 ± 0.7 vs. 6.2 ± 0.8), aroma (7.1 ± 0.9 vs. 5.6 ± 1.0), and texture (7.0 ± 0.5 vs. 5.7 ± 0.9) were rated more favorably for the shorter fermentation time. The 24 h fermented beverage exhibited a mild sourness balanced by a delicate creamy taste and is described as having a pleasant, slightly nutty aroma derived from chickpea water extract, which has a sensory quality suitable for the consumer.

It should be noted that the sensory evaluation in this study was conducted with a panel of 30 untrained consumers, which may limit the generalizability of the acceptability results to broader population groups. Future studies with larger, more diverse consumer panels and trained sensory assessors are recommended to further validate these findings.

#### 3.4.2. Determination of Acid Forming Activity in Beverages

The increase in titratable acidity and a significant decrease in pH due to the use of a combination of two bacteria, *Lactobacillus fermentum* SB-2 and *Lactobacillus sakei* SD-8, in a dairy beverage containing chickpea water extract is evident in the results. A pH of 3.5 with a titratable acidity of 107 °T after 24 h of fermentation indicates significant acid production, which increased to 164 °T with increasing time to 72 h. Even though the pH stayed at 3.5, which indicates a consistent concentration of hydrogen ions, the accumulation of organic acids persisted. According to the results in Table 9, we observed greater acidity in the fermented beverage containing chickpea water extract compared to the control, indicating increased metabolic activity in the presence of this water extract.

#### 3.4.3. Determining the Chemical Composition of Beverages

The fermented beverage’s composition was analyzed, revealing the following dry matter content: 14.3 ± 0.5% total solids, including 10.60 ± 0.30% protein, 0.82 ± 0.05% fat, 1.84 ± 0.15% carbohydrates, and 1.04 ± 0.05% ash. The production of this low-fat, nutrient-rich fermented dairy-plant mixture will satisfy consumer demand because of these qualities, which are also in accordance with recent research [44] and the regulatory requirements of the Eurasian Economic Union (EAEU) for products containing milk.

#### 3.4.4. Proteomic Studies of Fermented Beverages

**Figure 8:**
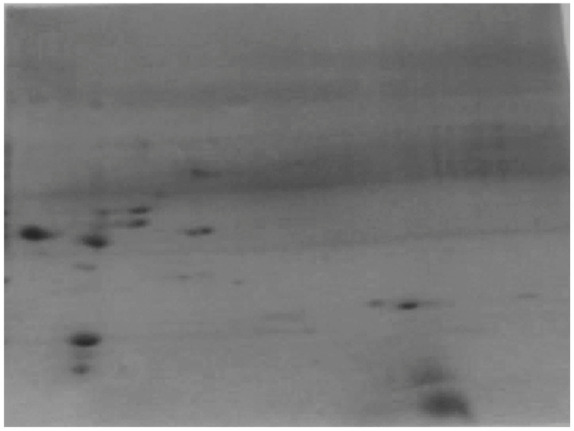
Two-dimensional electrophoresis of dairy beverage proteins with fermented chickpea water extract.

It was found that proteolysis of chickpea proteins by *L. fermentum* SB-2 and *L. sakei* SD-8 resulted in the formation of peptides with various potential biological activities (antitumor, antihypertensive, antituberculosis, antioxidant, antifungal, antibacterial) and angiotensin-converting enzyme (ACE) inhibitors. The samples also contained typical milk proteins, detectable on electrophoregrams (Figure 8). The identification results showed that these are: α-S1 casein phosphorylated at the 130S serine residue, a mixture of β-casein phosphorylated at 50S and a fragment of α-S1 casein, as well as a mixture of three proteins—β-lactoglobulin, a fragment of progestogen-associated endometrial protein and a fragment of β-casein.

## 4. Conclusions

In this study, the production of biologically active peptides through the fermentation of chickpea proteins and the production of milk beverage based on chickpea water extract were investigated. The use of the capacity of lactic acid bacteria to digest chickpea proteins and produce peptides with predicted bioactivities, including potential antioxidant, anticancer, antibacterial, antihypertensive, antifungal, antituberculosis and angiotensin-converting enzyme (ACE) inhibitors, which were determined by proteomic methods, was clearly seen in the experiments. Future investigations employing gas chromatography-mass spectrometry (GC-MS) analysis of volatile organic compounds (VOCs) could further elucidate the metabolic pathways responsible for the strain-dependent aroma profiles observed in this study, providing deeper insights into the flavor development mechanisms during chickpea-based beverage fermentation.

Beyond identifying peptide profiles, this study provides four distinct contributions to the field: (1) the first comprehensive proteolytic gene profiling (prtB, prtP, prtR, prtH) of ten LAB strains isolated from diverse traditional sources and their substrate-specific activity against chickpea proteins; (2) an integrated proteomic-bioinformatic workflow combining 2-DE, direct MALDI-TOF MS/MS (without tryptic digestion for short peptides), and multi-database prediction to identify 30 unique chickpea-derived peptides with diverse predicted bioactivities; (3) a direct application-oriented translation of screening results into a prototype fermented dairy-beverage (55% milk + 45% chickpea extract) compliant with EAEU technical regulations (TR CU 033/2013); and (4) the successful deposition of all ten newly isolated LAB strains in the All-Russian Collection of Industrial Microorganisms (VKPM), establishing a valuable biological resource for future functional food development.

Given that today, concerns about human health are taken into account and the interest in healthy beverages enriched with beneficial compounds has increased, and their cost-effectiveness is also considered to be important factors, the use of the results of this study will be very effective in promoting health in the food and pharmaceutical industries.

### Limitations

It is important to acknowledge that the biological activities reported for the peptides identified in this study are based on in silico predictions using bioinformatics databases (BIOPEP, AntiCP, AntiBP, AHTpin, ToxinPred, AntiFP, AntiTb). While these tools are valuable for preliminary screening and hypothesis generation, predicted activities do not always correlate with experimental in vitro or in vivo results, and their accuracy depends on the quality and completeness of reference data [45]. Therefore, the functional claims made herein should be interpreted as potential activities requiring experimental confirmation.

To address this limitation, our research group has initiated complementary experimental work (i) enzymatic hydrolysis of chickpea protein isolate followed by antioxidant capacity assessment (FRAP, DPPH, ORAC) and identification of active peptides such as SSSSPDIYIPQAGR [46]; (ii) molecular docking analysis confirming the binding potential of this peptide to oxidative stress-related targets [47]; and (iii) chemical synthesis of selected in silico-identified peptides from chickpea, rapeseed, and hemp proteins, with ongoing evaluation of their antioxidant, hypoglycemic, and hypolipidemic activities in cellular models under RSF project № 25-16-00178. These efforts represent a systematic transition from computational prediction to experimental validation, and their results will be reported in forthcoming publications.

Furthermore, it should be acknowledged that the bioactivity predictions reported herein are based on peptide sequences identified in the fermented extract prior to simulated gastrointestinal digestion. The stability of these peptides under gastric (pH 2.0, pepsin) and intestinal (pH 7.5, pancreatin) conditions, as well as their intestinal absorption potential, were not assessed in this study. Future work should incorporate in vitro digestion models (e.g., INFOGEST protocol) and Caco-2 cell transport assays to evaluate the bioaccessibility and bioavailability of the identified peptides.

## Figures and Tables

**Table 1:** Isolation sources of lactic acid bacteria strains used in this study.

№	Isolation Source	Species	Strain	Strain Deposition Number (VKPM) *
1	Dried bear meat	*Limosilactobacillus fermentum*	SB-2	B-14054
2	Dried reindeer meat	*Latilactobacillus sakei*	SD-8	B-14053
3	Homemade yogurt	*Levilactobacillus brevis*	VY-1	B-14052
4	Sauerkraut	*Pediococcus pentosaceus*	FC-9	B-14055
5	Homemade cheese	*Pediococcus pentosaceus*	FC-10	B-14056
6	Curdled milk	*Leuconostoc mesenteroides*	FM-4	B-14057
7	Pickle brine	*Lactiplantibacillus plantarum*	PC-7	B-14058
8	Cottage cheese	*Leuconostoc mesenteroides*	CH-5	B-14059
9	Asparagus (sample 1)	*Limosilactobacillus fermentum*	AS-3	B-14060
10	Asparagus (sample 2)	*Lacticaseibacillus paracasei*	CA-6	B-14061

* VKPM, All-Russian Collection of Industrial Microorganisms (Moscow, Russia). Strains were deposited under the indicated accession numbers.

**Table 2:** Primers used in this study.

№	Primers	Annealing Temperature	Primer Sequence	PCR Amplicon Length
1	PrtP700	56	GCTTGAATTCGTTGTCGCTGCGGTTGT	685 bp
2	PrtM700	56	GCATGAATTCAATGCACGATAAATGAG	685 bp
3	P15C	55	AACCAAATCTGATGTTG	560 bp
4	P06C	55	TTTCAGCGGAAGCAACT	560 bp
5	PRTB10	56	GGTGTTGCTCCTGATGCCCAGC	597 bp
6	PRTB20	56	CCCCGTTTAACAACTGCAAGTT	597 bp
7	Jp23	56	GCTTGGATAGTAGCGTTAGC	1034 bp
8	Jp25	56	GGTGAACAAACTGAAGACG	1034 bp
9	prti2	53	CAACACCGGGACCACGGTG	1052 bp
10	IP6Xba	53	CTGATCGTGGACGGTGTTGC	1052 bp

**Table 3:** Sensory aroma profiles of chickpea beverage fermented with different LAB strains.

№	Strain	Aroma Description
1	*Limosilactobacillus fermentum* SB-2	Sensorially neutral, Slightly sour, Fresh aroma lacking off-notes
2	*Latilactobacillus sakei* SD-8	Sensorially neutral, Slightly sour
3	*Levilactobacillus brevis* VY-1	Sharp lactic acidity, Kefir-like
4	*Pediococcus pentosaceus* FC-9	Fresh aroma lacking off-notes
5	*Pediococcus pentosaceus* FC-10	Sensorially disagreeable, Pungent
6	*Leuconostoc mesenteroides* FM-4	Pungent, sour
7	*Lactiplantibacillus plantarum* PC-7	Sensorially agreeable, Fresh aroma lacking off-notes
8	*Leuconostoc mesenteriodes* CH-5	Slightly sour
9	*Limosilactobacillus fermentum* AS-3	Sensorially disagreeable, Proteolytic off-odors
10	*Lacticaseibacillus paracasei* CA-6	Sensorially disagreeable, Pungent

**Table 4:** PCR detection of cell envelope proteinase (CEP) genes in 10 LAB strains.

Gene	*Lacticaseibacillus* *paracasei* CA-6	*Limosilactobacillus* *fermentum* AS-3	*Leuconostoc* *mesenteroides* CH-5	*Lactiplantibacillus* *plantarum* PC-7	*Leuconostoc* *mesenteroides* FM-4	*Pediococcus* *pentosaceus* FC-10	*Pediococcus* *pentosaceus* FC-9	*Limosilactobacillus* *fermentum* SB-2	*Latilactobacillus* *sakei* SD-8
prtP⁄prtM (685 bp)								+	
prtP (560 bp)	+	+		+			+		
prtB (597 bp)				+	+	+		+	+
prtH (1034 bp)	+	+					+		
prtR (1052 bp)		+	+		+	+			+

“+” indicates the presence of the target proteolytic gene in the respective strain; blank spaces indicate the absence of the gene.

**Table 5:** TNBS assay results: pH and L-leucine equivalents during chickpea beverage fermentation.

Sample	Time Fermentation, h	pH	Equivalents L-Leucine, mM	Δ Equivalents L-Leucine, mM *
*Limosilactobacillus fermentum* SB-2	0	6.14	10.56	0
	72	4.13	27.91	17.35
*Latilactobacillus sakei* SD-8	0	6.19	9.59	0
	72	3.47	25.72	16.13
*Levilactobacillus brevis* VY-1	0	6.15	10.64	0
	72	3.7	25.38	14.74
*Pediococcus pentosaceus* FC-9	0	6.13	13.34	0
	72	3.63	25.92	12.58
*Pediococcus pentosaceus* FC-10	0	6.2	12.68	0
	72	3.85	26.88	14.2
*Leuconostoc mesenteroides* FM-4	0	6.14	12.31	0
	72	3.87	22.84	10.53
*Lactiplantibacillus plantarum* PC-7	0	6.2	12.51	0
	72	3.47	15.89	3.38
*Leuconostoc mesenteroides* CH-5	0	6.13	11.53	0
	72	3.48	19.57	8.04
*Limosilactobacillus fermentum* AS-3	0	6.1	10.99	0
	72	3.23	14.74	3.75
*Lacticaseibacillus paracasei* CA-6	0	6.12	11.11	0
	72	3.3	14.83	3.72
Chickpea beverage without bacteria (Control)	0	6.2	10.91	0
	72	5.31–5.34	14.72	3.73

* Δ L-leucine equivalents—the difference in the amount of L-leucine at 0 and 72 h.

**Table 6:** Mass spectrometric identification of protein fractions in unfermented chickpea beverage.

№	Protein Name (Gene Symbol)	Numbers of Protein NCBI	S/M/C *	Mm/pI (Exp.) **	Mm/pI (Calcul.) **
1	Seed biotin-containing protein SBP65 (LOC101504303)	XP_004487170.1	290/29/59	71.0/5.40	71.3/5.97
2	Mixture of vicilin-like N-terminal fragment and seed storage protein At2g18540 (LOC101506263)	XP_004503582.1	321/48/42	70.0/4.90	82.5/5.78
3	N-terminal missing fragment provicilin-like (LOC101510367)+ hexoses	XP_004496703.1	91/32/56	43.0/9.10	64.6/6.61
4	N-terminal fragment of legumin A-like (LOC101489278)	XP_004493780.1	276/25/48	36.0/5.20	59.8/5.87
5	C-terminal missing legumin A-like fragment (LOC101489278) *** (1)	XP_004493780.1	249/25/42	36.0/5.00	59.8/5.87
6	C-terminal missing legumin-like fragment (leg) *** (1)	XP_027188788.1	211/21/32	28.0/5.00	56.7/5.97
7	C-terminal missing legumin-like fragment (leg) *** (1)	XP_027188788.1	173/20/33	29.0/5.10	56.7/5.97
8	Vicilin-like fragment (LOC101515515) *** (1)	XP_004493035.1	393/39/71	28.0/7.80	51.9/5.73
9	albumin-2-like (LOC101512722)+ Acetyl (Protein N-term)	NP_001351664.1	487/32/96	25.0/5.10	26.1/5.67
10	Legumin J-like fragment (LOC101501269) *** (1)	XP_004495100.1	144/25/52	23.5/4.90	60.3/5.50
11	N-terminal fragment of vicilin-like (LOC101505411) *** (1)	XP_004492829.1	110/21/34	24.0/7.70	51.1/6.10
12	Mixture of N-terminal vicilin-like fragment (LOC101515515) and P24 oleosin (LOC101509783)	XP_004493035.1 XP_004489219.1	106/9/30 78/6/43	20.0/8.00	51.9/5.73 20.7/7.85
13	C-terminal fragment of legumin A-like (LOC101489278) *** (1)	XP_004493780.1	120/13/24	20.0/9.20	59.3/5.87
14	C-terminal fragment of legumin-like (LEG) *** (1)	XP_027188788.1	360/23/32	20.0/9.40	56.2/5.97
15	C-terminal fragment of legumin J-like (LOC101501269)	XP_004495100.1	152/16/24	20.0/10.0	60.3/5.50

* Score/Matches/Coverage—Score, indicator of compliance (significant protein scores are ≥68, *p* < 0.05); the number of Matching peptides; Coverage (%) of the complete amino acid sequence of the protein by the peptide; ** Mm/pI (experimental)—estimates based on the electrophoretic mobility on two-dimensional electrophoresis; Mm/pI (calculated)—estimates made from amino acid sequence data based on the removal of the signal peptide but without other postsynthetic modifications (ExPASy Compute pI/Mw tool); *** msms—identification confirmed by tandem mass spectrometry with the number of sequenced tryptic peptides in brackets.

**Table 7:** Predicted potential bioactivities of chickpea-derived peptides identified by MALDI-TOF MS/MS.

№ Peptides	Protein Source	№ Positions in Amino Acid Sequence	Sequence Confirmed by MS/MS	*m*/*z*—Mass-to-Charge Ratio	Activity *
*Limosilactobacillus fermentum* SB-2, 24 h					
1	NADPH-dependent aldehyde reductase 1, chloroplastic-like XP_004494625.2	33–53	ASGEQKFPPQKQETQPGKEHA	2364.2	1, 3, 6
2	dehydrin DHN3 XP_004512937.1	22–47	IVQVDQYGNPINQSGVGMTGEAGRTF	2738.3	1, 6
3	late embryogenesis abundant protein D-34-like XP_004496718.1	1–23	MNQEQPRRHQADQDPIKYGDVLP	2777.4	1, 4, 6, 7
*Limosilactobacillus fermentum* SB-2, 36 h					
4	vicilin-like XP_004493035.1	144–156	LAIPVNRPGQFQS	1426.8	1, 6
*Latilactobacillus sakei* SD-8, 24 h					
5	2S albumin-like XP_004487601.1	30–51	EIPESCHKQLKSLNLKHCEKFL	2622.3	1, 3, 6, 7
6		30–52	EIPESCHKQLKSLNLKHCEKFLM	2753.4	1, 2, 6, 7
7		24–55	SKDEKEEIPESCHKQLKSLNLKHCEKFLMKRM	3884.9	1, 4, 6, 7
*Latilactobacillus sakei* SD-8, 36 h					
8	2S albumin-like XP_004487601.1	24–51	SKDEKEEIPESCHKQLKSLNLKHCEKFL	3338.6	1, 3, 4, 6, 7
*Levilactobacillus brevis* VY-1, 24 h					
9	vicilin-like XP_004493035.1	377–388	GFGINAQNNQRN	1332.6	6
10		376–388	LGFGINAQNNQRN	1445.7	1, 6, 7
11	2S albumin-like XP_004487601.1	131–143	LRCGITPPLGCDL	1355.7	1, 6
*Pediococcus pentosaceus* FC-9, 24 h					
12	2S albumin-like XP_004487601.1	131–147	LRCGITPPLGCDLSFDN	1490.7	1, 6
*Pediococcus pentosaceus* FC-9, 36 h					
13	vicilin-like XP_004493035.1	420–428	LLKNQRQSH	1123.6	1, 2, 4, 7
*Pediococcus pentosaceus* FC-10, 24 h					
14	vicilin-like XP_004493035.1	400–419	IQRPVKEVAFPGSAEEVDR	2127.1	1, 5, 6
*Pediococcus pentosaceus* FC-10, 36 h					
15	vicilin-like XP_004492829.1	328–346	KKEDEEEEEDRNVQVQRFQ	2435.2	1, 3, 5, 6
*Leuconostoc mesenteroides* FM-4, 24 h					
16	vicilin-like XP_004492829.1	395–420	VISQIQRPVKEVAFPGSAEEVDRLLK	2908.6	1, 2, 6, 7
17		387–405	FLAGEEDNVISQIQRPVKE	2172.1	1, 5, 6
18		328–358	KKEDEEEEEDRNVQVQRFQSKLSSGDVVVIP	3616.8	1, 5, 6
19		328–359	KKEDEEEEEDRNVQVQRFQSKLSSGDVVVIPA	3687.8	1, 5, 6
*Leuconostoc mesenteroides* FM-4, 36 h					
20	2S albumin-like XP_004487601.1	77–90	REEGLKENCCAQL	1490.7	1, 6, 7
*Lactiplantibacillus plantarum* PC-7, 24 h					
			–		
*Lactiplantibacillus plantarum* PC-7, 36 h					
21	2S albumin-like XP_004487601.1 P24 oleosin XP_004489219.1	24–52	SKDEKEEIPESCHKQLKSLNLKHCEKFLM	3469.7	1, 3, 4, 6, 7
22		155–189	GSVADVAGYVGQKTKDVGQKTKEVGQDIQAKAHET	3642.8	1, 6, 7
*Limosilactobacillus fermentum* AS-3, 24 h					
23	dehydrin DHN3 XP_004512937.1	2–20	SYNQGQYVDQTRRTDEYGN	2336.0	1, 6
*Limosilactobacillus fermentum* AS-3, 36 h					
24	vicilin-like XP_004493035.1 P24 oleosin XP_004489219.1	331–348	KEDEEEEEDRNVQVQRFQ	2307.1	1, 3, 5, 6
25		155–193	GSVADVAGYVGQKTKDVGQKTKEVGQDIQAKAHETKRST	4115.0	1, 6, 7
*Lacticaseibacillus paracasei* CA-6, 24 h					
26	vicilin-like XP_004493035.1 oleosin 16.4 kDa-like XP_004515879.1 late embryogenesis abundant protein 2 NP_001296579.1 NADPH-dependent aldehyde reductase 1, chloroplastic-like XP_004494625.2	384–391	NNQRNFLA	976.5	1, 6
27		2–17	AQPQRGDYYDNYQQHP	2022.0	1, 3, 6, 7
28		138–155	FGMTNDDQDKDHFPTNRH	2175.0	1, 6, 7
29		33–52	ASGEQKFPPQKQETQPGKEH	2293.2	1, 3, 6
*Lacticaseibacillus paracasei* CA-6, 36 h					
30	seed linoleate 9S-lipoxygenase-3 XP_004486857.1	190–201	LRGDGTGERKEW	1403.7	1, 2, 5, 6

* (1) anticancer, (2) antibacterial, (3) antihypertensive, (4) antifungal, (5) antituberculosis, (6) angiotensin-converting enzyme (ACE) inhibitors, (7) antioxidative. Activities are predicted using bioinformatic databases and require experimental validation.

**Table 8:** Frequency distribution of predicted bioactivities among identified chickpea-derived peptides.

Activity Type	Count	Percentage (%)
ACE Inhibitor	30	100%
AntiCP (anticancer)	27	90%
Antioxidative	17	56.7%
AHTpin (antihypertensive)	10	33.3%
AntiFP (antifungal)	6	20%
AntiTb (antituberculosis)	6	20%
AntiBP (antibacterial)	4	13.3%

**Table 9:** Acid-forming activity (pH and titratable acidity) during fermentation in beverages.

Sample	Fermentation Duration, h	Acidity		% Lactic Acid (0.009 × °T)
		pH	Titratable, °T	
A fermented beverage containing two microbial strains at 10^7^ CFU/mL	0	6.69	27	0.243%
	24	3.5	107	0.963%
	72	3.5	164	1.476%
Cow’s milk fermented with two strains at 10^7^ CFU/mL (control)	0	5.56	25	0.225%
	24	5.18	88	0.792%
	72	3.8	135	1.215%

## Data Availability

The original contributions presented in this study are included in the article/Supplementary Materials. Further inquiries can be directed to the corresponding author.

## Supplementary Materials

The following supporting information can be downloaded at https://www.mdpi.com/article/10.3390/foods15122249/s1, Figure S1: Spectra of strain identification using MALDI-TOF: (a) *Limosilactobacillus fermentum* SB-2, (b) *Latilactobacillus sakei* SD-8, (c) *Levilactobacillus brevis* VY-1, (d) *Pediococcus pentosaceus* FC-9, (e) *Pediococcus pentosaceus* FC-10, (f) *Leuconostoc mesenteroides* FM-4, (g) *Lactiplantibacillus plantarum* PC-7, (h) *Leuconostoc mesenteriodes* CH-5, (i) *Limosilactobacillus fermentum* AS-3, (j) *Lacticaseibacillus paracasei* CA-6.
