# Supplementary material for: The Potential for Bioactive Peptide Production in a Fermented Dairy Beverage Based on Chickpea Water Extract Using Proteolytic Lactic Acid Bacteria
Source: Foods. 2026 Jun 22;15(12):2249. doi: 10.3390/foods15122249 (PMC13298703; doi:10.3390/foods15122249)
Supplement: Supplementary file 1 [file foods-15-02249-s001.zip › foods-4313269-supplementary.pdf]

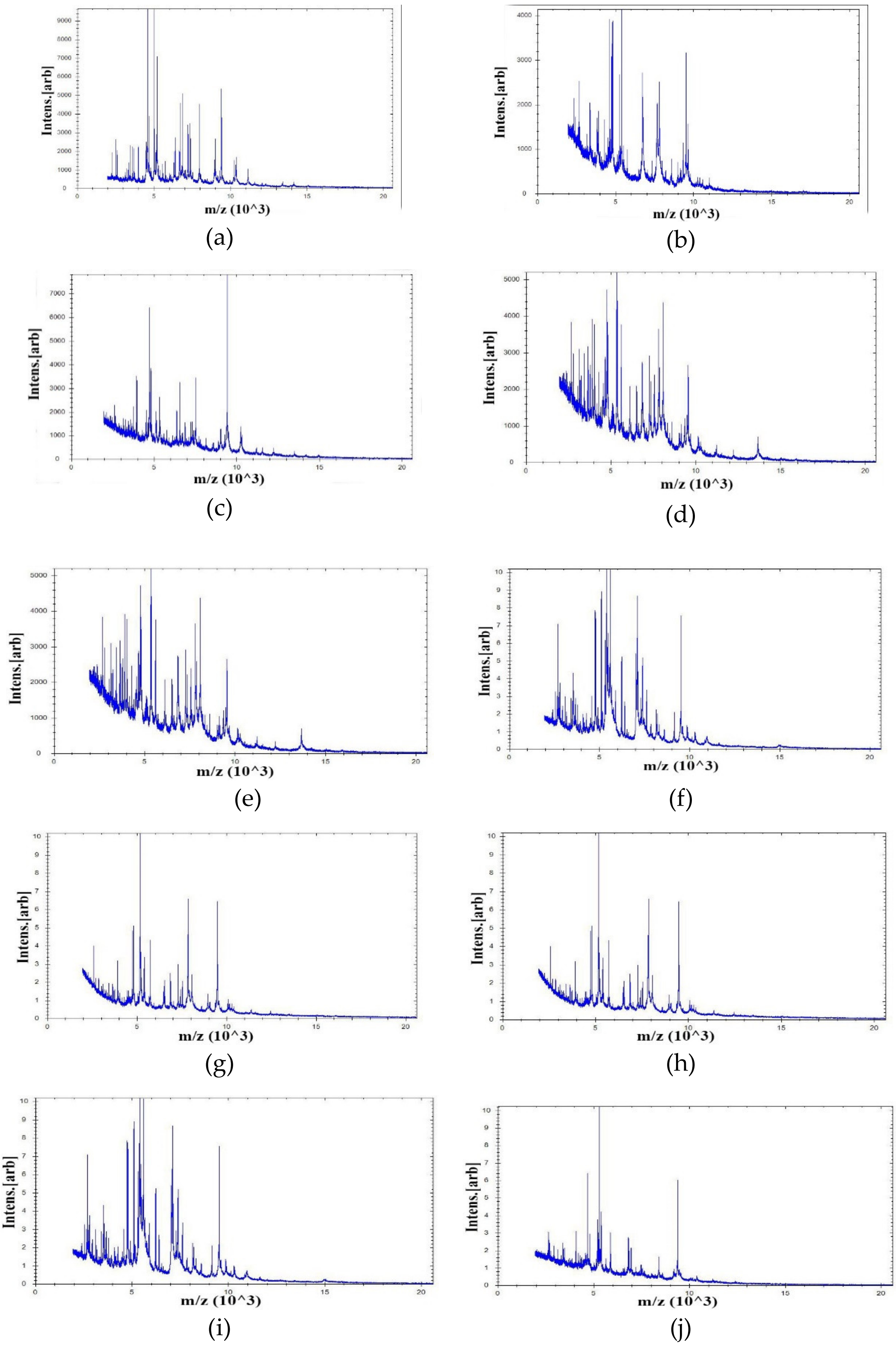

Figure S1. Spectra of strain identification using MALDI-TOF: (a) *Limosilactobacillus fermentum* SB-2, (b) *Latilactobacillus sakei* SD-8, (c) *Levilactobacillus brevis* VY-1, (d) *Pediococcus pentosaceus* FC-9, (e) *Pediococcus pentosaceus* FC-10, (f) *Leuconostoc mesenteroides* FM-4, (g) *Lactiplantibacillus plantarum* PC-7, (h) *Leuconostoc mesenteriodes* CH-5, (i) *Limosilactobacillus fermentum* AS-3, (j) *Lacticaseibacillus paracasei* CA-6.
